# Modeling and Analysis of Reservation Frame Slotted-ALOHA in Wireless Machine-to-Machine Area Networks for Data Collection

**DOI:** 10.3390/s150203911

**Published:** 2015-02-09

**Authors:** Francisco Vázquez-Gallego, Luis Alonso, Jesus Alonso-Zarate

**Affiliations:** 1 Centre Tecnològic de Telecomunicacions de Catalunya (CTTC), Castelldefels, Barcelona 08860, Spain; E-Mail: jesus.alonso@cttc.es; 2 Universitat Politècnica de Catalunya (UPC), Castelldefels, Barcelona 08860, Spain; E-Mail: luisg@tsc.upc.edu

**Keywords:** analysis, delay, energy consumption, reservation frame slotted-ALOHA, delta traffic

## Abstract

Reservation frame slotted-ALOHA (RFSA) was proposed in the past to manage the access to the wireless channel when devices generate long messages fragmented into small packets. In this paper, we consider an M2M area network composed of end-devices that periodically respond to the requests from a gateway with the transmission of fragmented messages. The idle network is suddenly set into saturation, having all end-devices attempting to get access to the channel simultaneously. This has been referred to as delta traffic. While previous works analyze the throughput of RFSA in steady-state conditions, assuming that traffic is generated following random distributions, the performance of RFSA under delta traffic has never received attention. In this paper, we propose a theoretical model to calculate the average delay and energy consumption required to resolve the contention under delta traffic using RFSA. We have carried out computer-based simulations to validate the accuracy of the theoretical model and to compare the performance for RFSA and FSA. Results show that there is an optimal frame length that minimizes delay and energy consumption and which depends on the number of end-devices. In addition, it is shown that RFSA reduces the energy consumed per end-device by more than 50% with respect to FSA under delta traffic.

## 1. Introduction

Machine-to-machine (M2M) communications are one of the fastest growing segments in information and communication technologies [1,2]. M2M networks will facilitate new and promising applications that will create a revolution in the way we run industries, cities, *etc.* However, the technology faces several significant challenges that need to be solved in order to get all of the potential of M2M. Among others, energy efficiency is crucial in the design of communication protocols for M2M networks that must operate for long periods of time with very limited access to energy sources and without human intervention.

In this paper, we focus on wireless M2M area networks [3], where a large group of hundreds or thousands of end-devices sense physical parameters from the environment and transmit the sensed information to a data concentrator, e.g., a gateway. Examples of such applications are: smart metering, where electricity, gas or water meters periodically transmit readings to a data-collector; asset tracking, where a radio frequency identification (RFID) reader sends requests to the tags, and they respond with their location and identification; and video surveillance, where a group of video cameras deployed in a building transmit still images every few minutes. In all of these data collection scenarios, end-devices remain in sleep mode for certain periods of time to save energy and wake up to transmit a burst of data packets when they are requested by the gateway. Therefore, the network changes abruptly from idle into saturation when all end-devices wake up to transmit simultaneously; this has been referred to in the literature as the delta traffic condition or batch arrival [4]. The contention process of a delta traffic condition starts when the gateway sends a request and finishes when all of the end-devices have succeeded in transmitting data. On the contrary, in steady-state conditions, it is assumed that end-devices generate data traffic following a given random distribution. Due to its mathematical tractability, stationary Poisson processes have been used in the past to model traffic generation. However, new applications and new communication scenarios, particularly posed by the Internet of Things, require a revision of existing models, including traffic generation models and their impact in communication protocols.

Since the total number of end-devices that may attempt to simultaneously get access to the channel can be potentially large under a delta traffic condition, an energy-efficient medium access control (MAC) protocol is needed to manage this traffic and to minimize the delay and energy waste due to collisions among end-devices and to idle listening periods devoted to carrier sensing. While time division multiple access (TDMA) allows every end-device to transmit without collisions, thus minimizing energy consumption, TDMA is not optimal in M2M networks with a large and dynamic number of end-devices, due to the delay and energy required to update the knowledge of the network topology and to create, maintain and distribute the network schedule. Contrarily, the simplicity and distributed operation of random access protocols [5,6], e.g., ALOHA or carrier sense multiple access (CSMA), make them ideal for simple and low-cost devices in M2M area networks with an unknown and dynamic number of end-devices. Many standards for wireless communications rely on random access protocols, such as the IEEE 802.15.4 [7] for low-rate wireless personal area networks, the wireless Meter-Bus (M-Bus) [8] for smart metering, the IEEE 802.11 [9] for wireless local area networks and the ISO/IEC18000-7 [10] for asset tracking.

The frame slotted ALOHA (FSA) protocol has been identified in the past as a good approach to handle the delta traffic in data collection scenarios due to its simplicity and good performance when optimally configured (e.g., [11–14]). As an example, FSA was adopted in the ISO/IEC 18000-7 standard, which is targeted at active RFID systems. In FSA, time is organized into frames divided into access slots. Each end-device randomly selects one slot of a frame to transmit data. The works in [11–13] show that the energy consumption of the RFID tags grows exponentially with the number of tags. The work in [14] also shows that there is an optimal frame length (*i.e.*, number of slots per frame) that minimizes the delay to collect data and the energy consumption of the entire network. This optimal frame length is equal to the number of end-devices. Unfortunately, those works consider that every end-device has just one data packet (e.g., identification, measurement, *etc.*) to transmit to the reader or data-collector. However, some other M2M data-collection applications, such as smart metering and video surveillance, for example, will require the transmission of longer messages from each end-device in every data collection round. These messages will be fragmented into smaller parts, which may require a new channel invocation to be transmitted.

The reservation FSA protocol (RFSA) was proposed in [15] to improve the performance of FSA when devices generate long messages with packet fragmentation. In RFSA, when an end-device succeeds in transmitting the first packet of a message in a given slot, that slot is reserved for that end-device in subsequent frames until the last packet of the message is sent. A number of research works have proposed the use of RFSA in satellite communications [16–19] and inter-vehicle communication [20–22]. As discussed into more detail later in Section 2, previous works focus on the throughput analysis of RFSA, assuming that the network is in steady-state conditions and consider that end-devices generate long messages following a given random distribution. Results show that RFSA outperforms FSA in terms of throughput. However, existing analytical models of RFSA do not evaluate the delay and energy consumption required to resolve the contention under delta traffic. Therefore, the analysis of RFSA under delta traffic conditions remains an open research question, which deserves attention for its application in data-collection scenarios. This is the main motivation for the work presented in this paper, which aims at filling this gap with the following contributions:

(1) We formulate an accurate theoretical model to calculate the average delay and the average energy consumption required to resolve the contention using RFSA in M2M networks under delta traffic and with fragmentation of long data messages. We validate the theoretical model with extensive computer-based simulations.
(2) We perform a comprehensive analysis and performance evaluation of RFSA and FSA to optimize delay and energy consumption. For this particular performance evaluation, we have considered radio transceivers in compliance with the IEEE 802.15.4 standard [7]. Results show better performance of RFSA for its application in low-complexity M2M data-collection networks.

The remainder of this paper is organized as follows. In Section 2, we describe the related work in order to motivate the contribution presented in this paper. In Section 3, we describe the system model and the operation of FSA and RFSA in data collection scenarios. In Section 4, we present the probabilistic analysis of RFSA based on absorbing Markov chains. Section 5 is devoted to the formulation of the analytical models to calculate the average delay and energy consumption for RFSA. In Section 6, we validate the models through computer simulation and evaluate the performance of both protocols in terms of delay and energy consumption. Finally, Section 7 concludes the paper.

## 2. Related Work

RFSA is an evolution of FSA to make it suitable when devices generate long messages divided into smaller packets or fragments. The concept of RFSA was originally proposed in [15] and later adapted to satellite communications [16–19] and inter-vehicle communication [20–22]. The contention process is composed of a sequence of time frames divided into slots. When an end-device successfully transmits a packet in a slot of a given frame, that particular slot is reserved for the same end-device in subsequent frames. As a result, the end-device has the equivalent of an assigned time division multiple access (TDMA) channel for as long as it has packets to send. The slots that are either empty or where a collision occurs remain available in subsequent frames for the contention of other devices, just as in FSA.

The work in [18] has studied the performance of RFSA in terms of average message delay and channel throughput in satellite communications. For the purpose of the analysis, the authors consider that each user (or end-device) generates messages according to a stationary Poisson process with a rate of λ messages per second, where each message consists of *υ* packets of fixed length, and *υ* is a random variable geometrically distributed with mean *ῡ*. Results show that RFSA adapts to the nature of the traffic. The maximum channel throughput of RFSA ranges from that of slotted-ALOHA when *ῡ* = 1, to that of fixed assigned TDMA channels when *ῡ* → ∞.

The work in [17,19] has also analyzed the dynamic behavior of RFSA with long messages. The author assumes that each user generates messages according to a geometrically-distributed process, and each message consists of a group of packets, whose number is geometrically distributed. A Markovian model is formulated to analyze the throughput versus average message delay trade-off characteristic. Results show that the performance of RFSA is very similar to slotted-ALOHA when the mean number of packets per message tends to one. However, RFSA outperforms slotted-ALOHA in terms of throughput and average message delay when the mean number of packets per message increases.

The work in [22] studied the performance of the RFSA protocol with priority (PR-ALOHA) proposed for inter-vehicle communication. Priority is introduced in RFSA by reserving specific time slots in the frame of RFSA exclusively for high priority traffic. Traffic is divided into either low or high priority (e.g., safety-related messages or multimedia). When a user generates a normal priority message, it may contend for any empty slot that is not high priority. When the message is of high priority, the user may contend for any empty slot, including the slots not reserved for high priority traffic. As could be expected, the simulation results show that as the number of slots reserved for high priority increases, the delay of the high priority messages decreases. However, the throughput of the low priority messages decreases, and their delay increases.

The work in [23] proposes the reservation-contention resolution diversity slotted ALOHA protocol (R-CRDSA) for satellite networks. R-CRDSA introduces the basic concept of CRDSA [24] in the RFSA protocol. In CRDSA, each user transmits several replicas of each packet in a frame using different randomly selected slots. The coordinator (e.g., satellite transponder) implements an interference cancellation (IC) algorithm that allows decoding data packets that were initially lost due to collision. When a packet is successfully decoded in a given slot, that slot is allocated to the successful user. This user will transmit the rest of the packets of its message in the allocated slot without any replicas in other slots. The authors assume long message arrivals at each user, where each message consists of a random number of packets with an arbitrary distribution. The results show that when the average number of packets per message is one, the performances of R-CRDSA and RFSA are very similar to CRDSA and slotted-ALOHA, respectively. As the average number of packets increases, R-CRDSA achieves the highest throughput. In addition, the differences in throughput between R-CRDSA and RFSA decrease as the mean number of packets per message increases.

To the best of our knowledge, existing performance evaluations of RFSA have been done in steady-state conditions, assuming that devices generate long messages following a random distribution, typically Poisson. However, the performance of RFSA in the case of delta traffic conditions remains an open research question.

## 3. System Model and Medium Access Control Protocols

### 3.1. System Model

We consider a single-hop wireless network composed of one coordinator surrounded by *n* end-devices in its transmission range. The coordinator collects data from the end-devices by periodically initiating data collection rounds (DCR). Each DCR is initiated when the coordinator broadcasts a request for data (RFD) packet. In every DCR, each end-device has a number *L* of data packets ready to transmit to the coordinator, where *L* is an exponentially distributed random variable. The value of *L* is independent of other end-devices and has a new realization in every DCR. The assumption of exponentially-distributed message lengths is common in the analysis of communication networks [25] and has been traditionally used to model random data processes related to different sources of data.

We assume that the end-devices are in a low-power listening mode [26] in which they periodically wake up and turn on the radio transceiver for a short period of time to detect communication requests from the coordinator. We assume that all of the end-devices are synchronized and listen to the channel when the coordinator transmits an RFD. After decoding an RFD, the end-devices start contending for the channel to transmit their *L* data packets within a sequence of time frames. Each frame is composed of a fixed number *m* of slots. An end-device transmits each one of its data packets in one slot of one frame according to the rules of the adopted MAC protocol, as detailed in Section 3.2. The data packets have a fixed size and fit within the duration of a slot.

The end-devices transmit without performing carrier sensing; thus, a given slot can be in one of three states: (i) empty, *i.e.*, no data packet has been received by the coordinator in the slot; (ii) success, *i.e.*, a data packet has been received and decoded by the coordinator; or (iii) failure, *i.e.*, one or more data packets have been transmitted in the slot, but none have been decoded by the coordinator. This later case includes both collisions and channel errors. At the end of each frame, the coordinator transmits a feedback packet (FBP) to inform about the data packets successfully received in every frame. Once an end-device succeeds in transmitting its *L* data packets, it becomes inactive for the remainder of the DCR to save energy.

In order to focus on the contention process, we consider a communication scenario where all packets are always transmitted without transmission errors induced by the wireless channel. In addition, we assume that when two or more data packets collide, none of them can be decoded by the coordinator, *i.e.*, there is no capture effect. The inclusion of the capture effect and transmission errors, and their impact on the overall network performance, constitutes part of our ongoing and future work.

Regarding energy consumption, the coordinator and end-devices can be in five different modes of operation: (i) transmitting a packet; (ii) receiving; (iii) idle listening; (iv) standby; or (v) sleeping. The associated power consumptions are *ρ_tx_*, *ρ_rx_*, *ρ_σ_*, *ρ_stby_* or *ρ_sleep_*, respectively. The model is general for any power-saving mechanism and any value of the power consumptions in different modes of operation. We assume that the energy required to switch between inactive (*i.e.*, standby, sleep) and active modes (*i.e.*, transmitting, receiving, idle listening) is negligible. In sleep mode, the radio interface is fully disabled, and thus, end-devices consume the lowest power consumption.

### 3.2. MAC Protocols

In this section, we describe the MAC protocols considered in this paper: FSA and RFSA. Although we focus on the analysis of RFSA, FSA is also described in order to make the paper self-contained.

#### 3.2.1. Frame Slotted-ALOHA

**Figure 1.**
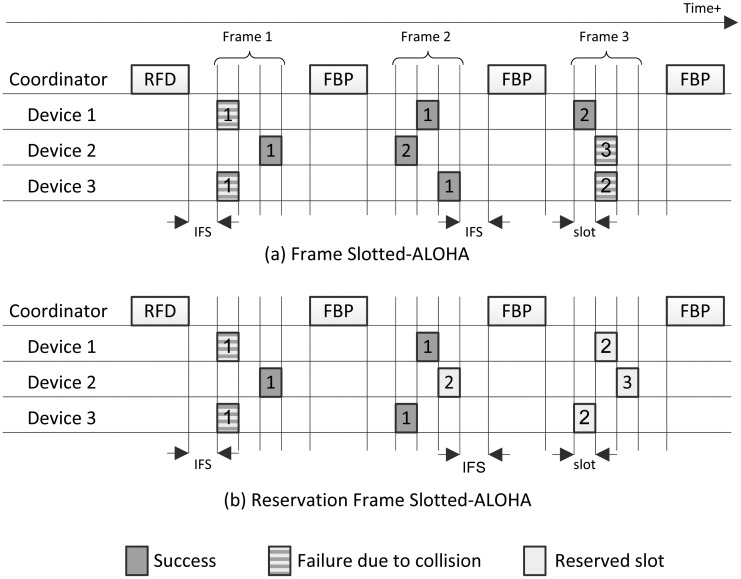
Example of a data collection round considering *n* = 3 end-devices and *m* = 3 slots per frame using: (**a**) frame slotted-ALOHA (FSA); and (**b**) reservation FSA (RFSA). The numbers inside the slots indicate the sequence number of the data packet transmitted by each end-device.

An example of a DCR using FSA is depicted in Figure 1a. In every frame, each end-device randomly selects one of the *m* slots to transmit one of its *L* data packets. At the end of each frame, the coordinator broadcasts an FBP to inform about the state of the *m* slots: empty, success or failure. When an end-device succeeds in transmitting a data packet, it randomly selects one of the *m* slots of subsequent frames to transmit the next data packet in its sequence of *L* packets. This process is repeated, frame after frame, until all of the data packets are successfully transferred to the coordinator, thus contending independently for each data packet.

The example of Figure 1a shows FSA with *n* = 3 contending end-devices and *m* = 3 slots per frame. In Frame 1, all end-devices transmit their first data packet. End-devices 1 and 3 collide in Slot 1, and End-device 2 succeeds in transmitting its first packet in Slot 3 of Frame 1. In Frame 2, End-devices 1 and 3 transmit again their first packet, and End-device 2 transmits its second packet in Slot 1. All end-devices succeed in Frame 2. In Frame 3, End-devices 1 and 3 transmit their second packet, and End-device 2 transmits its third packet: End-device 1 succeeds in Slot 1, and End-devices 2 and 3 collide in Slot 2. The process continues after Frame 3, until all end-devices succeed in transmitting their *L* packets to the coordinator.

#### 3.2.2. Reservation Frame Slotted-ALOHA

An example of a DCR using RFSA is depicted in Figure 1b. In every frame, each end-device randomly selects one slot to transmit the first of its *L* data packets. When an end-device succeeds in transmitting its first data packet, the slot with success is reserved to that end-device for the next *L* − 1 frames to transmit its other data packets. At the end of each frame, the coordinator broadcasts an FBP to inform about the state of the *m* slots: empty, success or failure; and free or reserved. The end-devices that are still contending to transmit their first data packet can only select a slot among the free slots. When an end-device has transmitted the complete sequence of *L* data packets, its reserved slot becomes free again in order to be used by other contending end-devices in subsequent frames. An end-of-message flag in the header of the last packet indicates that an end-device releases his reserved slot. These processes are repeated, frame after frame, until the coordinator is able to decode the *L* data packets from every end-device.

The example of Figure 1b shows RFSA with three contending end-devices and three slots per frame. In Frame 1, End-device 2 succeeds in transmitting its first packet in Slot 3. Therefore, Slot 3 is reserved for End-device 2 for the transmission of its sequence of data packets in subsequent frames. Since End-devices 1 and 3 collide in Frame 1, they randomly select a new slot in Frame 2 (among the free Slots 1 and 2) to transmit their first packet. End-devices 1 and 3 succeed in transmitting their first data packet in Slots 2 and 1 of Frame 2, respectively. Therefore, Slots 1, 2 and 3 are reserved for End-devices 3, 1 and 2, respectively, for the transmission of their data packets. The process continues after Frame 3, until every end-device has transmitted its *L* packets to the coordinator.

Note that in both FSA and RFSA protocols, as shown in Figure 1, a guard time, called the inter-frame space (IFS), is left between reception and transmission to compensate for propagation, processing and turn-around times to switch the end-devices' radio transceiver between reception and transmission modes.

## 4. Analysis of Reservation Frame Slotted-ALOHA

In this section, we analyze the contention process in a data collection round using RFSA. We propose an absorbing Markov chain to model the evolution of the number of contending end-devices and the number of free slots within the contention process. The proposed Markov chain is inspired by existing works in data collection scenarios [14,27]. In order to build the transition matrix that characterizes the Markov chain, we derive the probability that one or more end-devices succeed in transmitting their first data packet and the probability that a reserved slot becomes free in a given frame. Finally, we derive the average number of frames in which the process remains in every state, which is used later in Section 5 to formulate the delay and energy models for RFSA.

### 4.1. Discrete-Time Absorbing Markov Chain

**Figure 2.**
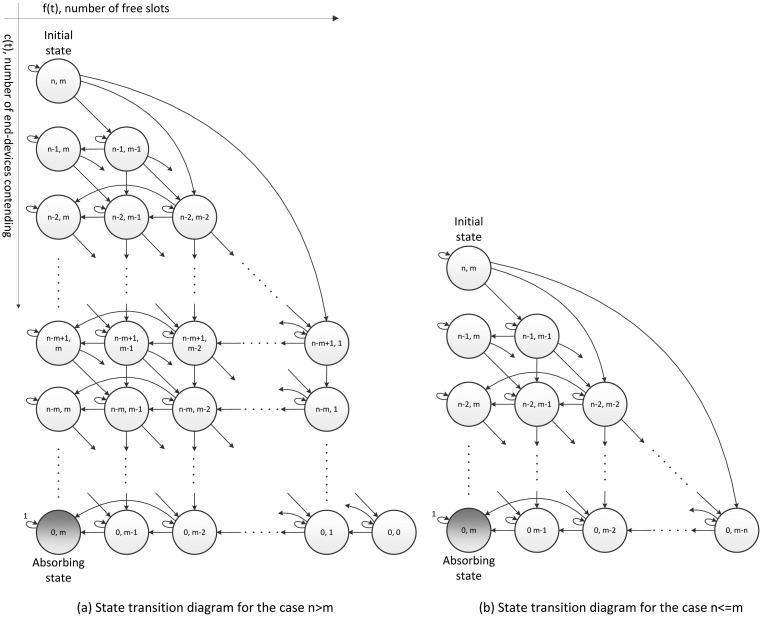
Generalized state transition diagram of the absorbing Markov chain of RFSA: (**a**) for the case in which the number *n* of end-devices is greater than the number *m* of slots; and (**b**) for the case in which the number *n* of end-devices is lower or equal to the number *m* of slots. Some transitions have been intentionally omitted for ease of understanding of the figure.

The contention process of RFSA can be modeled with a discrete-time absorbing Markov chain. The generalized state transition diagram is depicted in Figure 2, respectively, for the case where the number *n* of end-devices is greater than the number *m* of slots and for the case where the number *n* of end-devices is lower or equal to the number *m* of slots.

Each state of the chain is defined by {*c*(*t*), *f*(*t*)}, where *c*(*t*) ∈ {0,1, …, *n*} and *f*(*t*) ∈ {0, 1, …, *m*} are stochastic processes that represent the number of end-devices that are contending to transmit their first data packet and the number of free slots at time *t*, respectively. Then, the number of reserved slots at time *t* can be represented by *r*(*t*) = *m* − *f*(*t*). A discrete time scale is adopted, *i.e.*, *t* and *t* + 1 correspond to the beginning of two consecutive frames.

The process starts at *t* = 1, where *c*(*t*) = *n* and *f*(*t*) = *m*, *i.e.*, the initial state is (*n, m*), and it finishes at the absorbing state (0, *m*). By observing the state transition diagrams depicted in Figure 2, the total number *K* of states can be calculated as:

(1) $$K=\{\begin{array}{ll} \left(\frac{m}{2}\right)(m+1)+(n-m+1)m+1, & if(n>m)\\ \left(\frac{n+1}{2}\right)(n+2), & if(n\leq m) \end{array}$$

The process changes from state (*c_i_*, *f_i_*) to state (*c_j_*, *f_j_*) in one-step, with *i, j* ∈ {1, 2, …, *K*}, when the following two conditions are met:

Condition 1: A number of contending end-devices succeed in transmitting their first data packet, denoted by *S_ij_*, which can be expressed as the difference between the number of contending end-devices in both states,
  (2) $$S_{ij}=\{\begin{array}{ll} c_{i}-c_{j}, & if(c_{i}>c_{j})\\ 0, & \text{otherwise} \end{array}$$
  and *S_ij_* ∈ {0, 1, …, max (*S_ij_*)}, where max (*S_ij_*) is the maximum number of contending end-devices that can succeed in one frame. Formally, max (*S_ij_*) can be defined as:
  (3) $$\max(S_{ij})=\{\begin{array}{l} 0,\\ f_{i}-1\\ c_{i}, \end{array}\begin{array}{r} if(f_{i}=1)\text{and}(c_{i}>1)\\ if(c_{i}>f_{i})\\ if(c_{i}\leq f_{i}) \end{array}$$
Condition 2: A number of reserved slots become free, denoted by *F_ij_* ∈ {0,1,…, (*m* − *f_i_*)}, which can be expressed as the sum of the difference between the number of free slots in both states (*f_j_* − *f_i_*), plus the difference between the number of contending end-devices in both states (*i.e., S_ij_*),
  (4) $$F_{ij}=\{\begin{array}{cc} (f_{i}-f_{j})+S_{ij}, & if(f_{i}<m)and(f_{i}\leq f_{j})\\ 0, & \text{otherwise} \end{array}$$

Then, we can formulate the one-step transition probability *p_ij_* from state (*c_i_*, *f_i_*) to state (*c_j_, f_j_*) as follows:

(5) $$p_{ij}=\{\begin{array}{ll} P_{s}(S_{ij},c_{i},f_{i})\left(\begin{array}{r} m-f_{i}\\ F_{ij} \end{array}\right){p_{r}}^{F_{ij}}{\left(1-p_{r}\right)}^{m-f_{i}-F_{ij}}, & if(c_{j}-\max(S_{ij})\leq c_{j}\leq c_{i})\text{and}(f_{j}\leq m-c_{i}+c_{j})\\ 0, & if(c_{j}>c_{i})or(c_{j}<c_{i}-\max(S_{ij}))or(f_{j}>m-c_{i}+c_{j})\\ 1-\underset{i\neq j}{\text{∑}}p_{ij}, & if(c_{i}=c_{j}\neq 0)\text{and}(f_{i}=f_{j}\neq m)\\ 1, & if(c_{i}=c_{j}=0)\text{and}(f_{i}=f_{j}=m) \end{array}$$

where *P_s_*(*S_ij_*, *c_i_*, *f_i_*) is the probability that there exists a number *S_ij_* of end-devices that succeed in transmitting their first data packet in a given frame with *f_i_* free slots having *c_i_* contending end-devices, and *p_r_* is the probability that a reserved slot is released (*i.e.*, a slot becomes free) when an end-device finishes transmitting its *L* data packets. We derive both probabilities *P_s_*(*S_ij_*, *c_i_*, *f_i_*) and *p_r_* later in Section 4.2.

The rationale for the transition probability is the following. In the first condition, *S_ij_* end-devices succeed in transmitting their first data packet to the coordinator, and *F_ij_* slots become free among the (*m* − *f_i_*) slots reserved. In the second condition, the number of contending end-devices increases, *i.e.*, *c_j_* > *c_i_*, it decreases below its lower bound, *i.e.*, *c_j_* < *c_i_* − max (*S_ij_*), or the number of free slots increases above its upper bound, *i.e.*, *f_j_* > *m* − *c_i_* + *c_j_*. The three options are impossible and have therefore zero probability. In the third condition, no new end-devices succeed in transmitting their first data packet, *i.e.*, *S_ij_* = 0, and the process is not yet absorbed, *i.e.*, *c_i_* = *c_j_* ≠ 0 and *f_i_* = *f_j_* ≠ *m*. In the fourth condition, the transition probability is one, because the process has finished and remains in the absorbing state (0, *m*).

### 4.2. Probabilities of Success and Release

According to [27], the probability *P_s_*(*S_ij_*, *c_i_*, *f_i_*) that *S_ij_* contending end-devices succeed in transmitting their first data packet in a frame, having *f_i_* free slots and *c_i_* contending end-devices, can be formulated as:

(6) $$P_{s}(S_{ij},c_{i},f_{i})=\frac{\left(\begin{array}{c} f_{i}\\ S_{ij} \end{array}\right)\overset{S_{ij}-1}{\underset{k=0}{\text{∏}}}\left(c_{i}-k)G(f_{i}-S_{ij},c_{i}-S_{ij}\right)}{{f_{i}}^{c_{i}}}$$

where:

(7) $$G(T,t)=T^{t}+\sum_{k=1}^{t}\left\{{\left(-1\right)}^{k}\cdot \prod_{j=0}^{k-1}\{(t-j)(T-j)\}{\left(T-k\right)}^{t-k}\frac{1}{k!}\right\}$$

with *T* = *f_i_* − *S_ij_* and *t* = *c_i_* − *S_ij_*.

A reserved slot becomes free when one end-device has transmitted all of its *L* data packets to the coordinator. Then, since *L* is an exponentially-distributed random variable, the probability that a reserved slot is released in a given frame can be expressed as:

(8) $$p_{r}=\frac{1}{L}$$

where *L̄* is the mean of the exponential distribution of *L.*

### 4.3. Average Number of Frames in the Transient States

In order to obtain the average time (in frames) in which the process remains in every transient state of the chain, we first order the *K* states as {*X*_1_, *X*_2_, …, *X_k_*}, where *X_i_* = (*c_i_*, *f_i_*). Being *X_k_* = (0, *m*) the absorbing state and the other the transient states, with *X*_1_ = (*n, m*), we can represent the absorbing Markov chain as a *K* × *K* transition matrix *P* defined as:

(9) $$P=\left[\begin{array}{cc} Q & R\\ 0 & 1 \end{array}\right]$$

where *Q* is a (*K* − 1) × (*K* − 1) matrix that contains the transition probabilities between transient states (*i.e.*, *p_ii_* ≠ 1 with *i, j* < *K*), *R* is a (*K* − 1) × 1 non-zero column vector that contains the transition probabilities from the transient states to the absorbing state (*i.e.*, *i* < *K* and *j* = *K*), one is the transition probability at the absorbing state (*i.e.*, *p_ii_* = 1 with *i* = *K*) and zero is a 1 × (*K* − 1) row vector of zeros.

The fundamental matrix *N* of *P* can be defined as:

(10) $$N={\left(I-Q\right)}^{-1}$$

where each element *n_ij_* of *N* is equal to the average number of frames that the process remains in the transient state *X_j_* (*i.e.*, with *c_j_* contending end-devices and *f_j_* free slots for *n_ij_* consecutive frames) given that it started in the transient state *X_i_* with *i, j* = {1, …, (*K* − 1)}.

The time of absorption, denoted by *t_absorption_*, is defined as the average number of frames until the process is absorbed (*i.e.*, it reaches state *X_K_*) when it started in state *X*_1_. The time of absorption can be expressed as:

(11) $$t_{\text{absorption}}=N\cdot c=\sum_{j=1}^{K=1}n_{1j}$$

where *c* is a (*K* − 1) × 1 column vector with all ones. The values of *n*_1_*_j_* with *j* = {1, …, (*K* − 1)} are used in Section 5 to formulate the theoretical models of delay and energy consumption using RFSA.

## 5. Delay and Energy Models

In this section, we use the analysis presented in the previous section to formulate the calculation of the average delay and energy consumption in a data collection round using the RFSA protocol described in Section 3.2.

The average delay, expressed in seconds and defined as the time elapsed since the data collection round starts until all end-devices succeed in transmitting their *L* data packets to the coordinator, can be formulated as:

(12) $$\bar{T_{CR}}=t_{\text{absorption}}(mT_{\text{SLOT}}+2T_{\text{IFS}}+T_{\text{FBP}})$$

where *T_SLOT_*, *T_IFS_* and *T_FBP_* are the duration of a slot, an IFS and the time of transmission of an FBP packet, respectively, and *t_absorption_* is the average number of frames that the process needs to be absorbed. Indeed, the process starts at state *X*_1_ = (*n, m*) and finishes when it reaches state *X_K_* = (0, *m*).

The average energy consumed by the coordinator is denoted by $\bar{E_{\text{coord}}}$ . The coordinator executes the following operations in every frame of the process: (i) listens to the channel for the *m* slots of each frame to receive incoming data packets; (ii) remains in idle mode for the duration of two IFS; and (iii) transmits the FBP after each frame. Therefore, the average energy consumption of the coordinator can be formulated as:

(13) $$\bar{E_{\text{coord}}}=t_{\text{absorption}}(m\rho_{rx}T_{\text{SLOT}}+2\rho_{\sigma }T_{\text{IFS}}+\rho_{tx}T_{\text{FBP}})$$

The average energy consumed by all of the end-devices is denoted by $\bar{E_{n-ed}}$. Since we consider a homogeneous network and the MAC rules are identical to all of the end-devices, the average energy consumed per end-device can be considered as symmetrical and be expressed as $\bar{E_{1-ed}}=\bar{E_{n-ed}}/n$.

An end-device that has not succeeded yet in transmitting all of its *L* data packets executes the following operations in each frame: (i) transmits a data packet in one of the *m* slots; (ii) switches to standby for the other *m* − 1 slots; (iii) stays in idle mode for two IFS; and (iv) receives the FBP. Thus, the energy consumed by one end-device in a frame where it transmits a data packet can be expressed as:

(14) $$E_{tx\_\text{data}}^{\text{frame}}=\rho_{tx}T_{\text{SLOT}}+(m-1)\rho_{\text{stby}}T_{\text{SLOT}}+2\rho_{\sigma }T_{\text{IFS}}+\rho_{rx}T_{\text{FBP}}$$

An end-device that has succeeded in transmitting its *L* data packets in a given frame will sleep in subsequent frames until the data collection round finishes. Thus, the energy consumed by one end-device in a frame where it does not transmit a data packet can be expressed as:

(15) $$E_{\text{sleep}}^{\text{frame}}=\rho_{\text{sleep}}(mT_{\text{SLOT}}+2T_{\text{IFS}}+T_{\text{FBP}})$$

The average energy consumed by all end-devices can be formulated as:

(16) $$\bar{E_{n-ed}}=\sum_{j=1}^{K-1}\left\{n_{1j}E_{j}^{\text{frame}}\right\}$$

where *n*_1_*_j_* is the average number of frames that the process remains in each transient state *X_j_* and $E_{j}^{\text{frame}}$ is the energy consumed by all end-devices in one frame being in state *X_j_*. Then, $\bar{E_{n-ed}}$ can be expressed as:

(17) $$\bar{E_{n-ed}}=\sum_{j=1}^{K=1}\left\{n_{1j}\left(N_{j}^{tx}E_{tx\_\text{data}}^{\text{frame}}+N_{j}^{\text{sleep}}E_{\text{sleep}}^{\text{frame}}\right)\right\}$$

where $N_{j}^{tx}=c_{j}+(m-f_{j})$ and $N_{j}^{\text{sleep}}=(n-N_{j}^{tx})$ are the number of end-devices that transmit a data packet and the number of end-devices that sleep, respectively, when the process is in state *X_j_*. Recall that *c_j_* and *f_j_* are the number of contending end-devices and the number of free slots in state *X_j_*, respectively, and *n* is the total number of end-devices that transmit data to the coordinator.

In the next section, we validate the accuracy of the delay and energy models for RFSA and evaluate the delay and energy performance for different network configurations using RFSA and FSA.

## 6. Model Validation and Performance Evaluation

In this section, we use the theoretical models formulated in Section 5 to calculate the average delay and energy consumption for RFSA under delta traffic conditions. We first describe the considered scenario. Then, we analyze how the delay and energy consumption are influenced by the number of slots per frame and the number of end-devices. We discuss the numerical results and determine the criteria to minimize the average delay and energy consumption. In addition, we compare the performance for RFSA and FSA.

### 6.1. Scenario

The system parameters used to validate the analytical models and to evaluate the performance are summarized in Table 1. They have been selected according to the IEEE 802.15.4 standard [7] and from the specifications of the CC2520 radio transceiver [28], typically used in M2M networks. We have considered that each end-device has a number *L* of data packets with a payload of 114 bytes ready to transmit in every data collection round, where *L* is exponentially distributed with mean *L̄* = 50 data packets per end-device. The length of the FBP payload has been set to attach two bits per slot that inform about the status of each slot. All of the packets are composed of a physical layer preamble, a MAC header, a payload and a cyclic redundancy code (CRC) of two bytes for error control.

We have validated the proposed theoretical models by means of computer-based simulations. A data collection round is simulated in MATLAB using a while loop in which all of the operations performed within one contention frame are simulated in each loop iteration. We use a 1 × *n* vector of packet counters to simulate the number of data packets that must be transmitted by each end-device. Each counter is set at the beginning of the simulation to the number *L* of data packets for each end-device and decreases when a new data packet is transmitted successfully in one loop iteration. We use a 1 × *n* vector to simulate the number of slot where each end-device transmits a data packet in one frame. In every iteration of RFSA, we generate a uniformly-distributed random number for each contending end-device to simulate the transmission of the first of its *L* data packets in a randomly-selected slot among the non-reserved slots. We use a 1 × *m* vector to simulate the transmission of a feedback packet from the coordinator that informs about the state of the *m* slots in every frame: (i) empty, if the slot has not been selected by any end-device; (ii) success (reserved), if the slot has been selected by one end-device and is reserved until the packet counter of the end-device is equal to zero, i.e., it finishes the transmission of its *L* data packets; (iii) failure (collision), if the slot has been selected by two or more end-devices; and (iv) released, if the slot becomes free again in order to be selected by other contending end-devices in subsequent iterations. The while loop is repeated until all of the packet counters are equal to zero.

The analytical results have been compared to the simulation within a range of frame lengths (*i.e.*, number of slots per frame) and for different numbers of end-devices. The results show a tight match between analysis and simulation in all tested cases, thus validating the correctness of the analyses. Results for FSA have also been obtained through computer-based simulations using MATLAB. The results of 1000 simulation samples have been averaged for each test case.

### 6.2. Criteria to Minimize Delay and Energy Consumption

**Figure 3.**
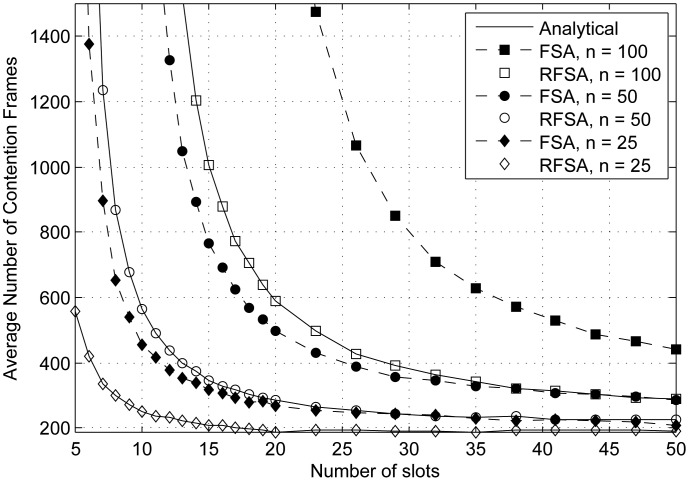
Average delay (in frames) required to terminate a data collection round over the frame length.

The average delay (*t_absorption_*) required to terminate a data collection round using RFSA and FSA, expressed in the average number of frames, is represented in Figure 3 as a function of the frame length (from five to 50 slots per frame). We have considered *n* = 25, 50 and 100 end-devices.

As could be expected, the results show that the value of *t_absorption_* decreases exponentially and tends to a constant value when the frame length increases. Indeed, the longer the frame length, the lower the probability of collision among end-devices and the higher the number of successful transmissions per frame. As can be observed, the value of *t_absorption_* is considerably greater in FSA than in RFSA. The fact that, in RFSA, an end-device that succeeds in transmitting its first data packet reserves the slot with success for the next *L* − 1 frames leads to a lower probability of collision in subsequent frames, since the number of contending end-devices is also reduced, yielding a shorter delay. The differences between the value of *t_absorption_* using RFSA and FSA become more apparent when the number of end-devices is higher or when the frame length is shorter, and thus, contention is greater in each frame.

**Figure 4.**
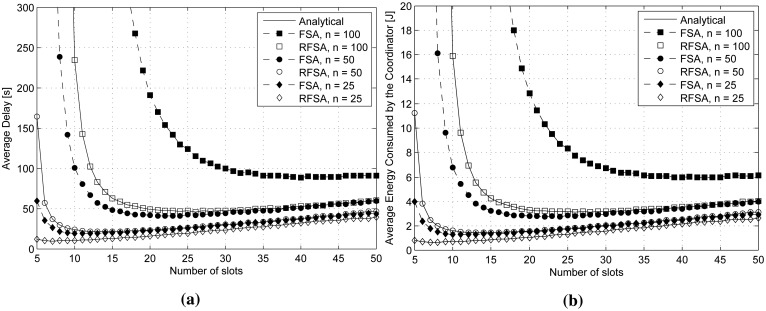
(**a**) Average delay (in seconds) required to terminate a data collection round over the frame length; and (**b**) average energy consumed by the coordinator in a data collection round over the frame length.

The average delay $\left(\bar{T_{CR}}\right)$, expressed in seconds, and the average energy consumed by the coordinator in a data collection round $\left(\bar{E_{\text{coord}}}\right)$ are represented in Figure 4, respectively. They have been evaluated as a function of the frame length (from five to 50 slots) for RFSA and FSA considering *n* = 25, 50 and 100 end-devices. It is worth noting that, as could be expected from the delay and energy models of RFSA, the values of $\bar{T_{CR}}$ in Equation (12) and $\bar{E_{\text{coord}}}$ in Equation (13) are highly correlated, showing a similar trend.

As can be observed in Figure 4, $\bar{T_{CR}}$ and $\bar{E_{\text{coord}}}$ tend to infinity in both RFSA and FSA when the frame length is lower than *n*/8 and *n*/4, respectively This is a direct consequence of the very high value of *t_absorption_*, due to the high probability of collision among the end-devices when the frame length is very low. In addition, the values of $\bar{T_{CR}}$ and $\bar{E_{\text{coord}}}$ in FSA are always greater than in RFSA. This is due to the fact that the values of *t_absorption_* are considerably greater in FSA, as shown in Figure 3.

There exists an optimum frame length *m_opt_* that minimizes the value of both $\bar{T_{CR}}$ and $\bar{E_{\text{coord}}}$. The value of the optimum frame length has been obtained numerically. First, we have computed the average delay and the average energy consumption over the frame length, and then, we have searched for the frame length that provides the lowest values of delay and energy consumption. We have repeated the process for different values of the number of end-devices in order to confirm the validity of the optimum value, which depends on the number of end-devices, being *m_opt_* ≃ *n*/5 for RFSA and *m_opt_* ≃ *n*/2 for FSA. For example, when *n* = 100 end-devices, we have minimum average delay, $\bar{T_{CR}}≃50\text{s}$, and minimum average energy consumption of the coordinator, $\bar{E_{\text{coord}}}≃3.2\text{J}$, at *m* ≃ 20 slots when using RFSA; and $\bar{T_{CR}}≃90\text{s}$ and $\bar{E_{\text{coord}}}≃6\text{J}$ at *m* ≃ 50 slots when using FSA. Therefore, RFSA provides a delay reduction and energy savings of approximately 45% with respect to FSA when using optimum frame lengths in both access protocols.

For frame lengths greater than *m_opt_*, the values of $\bar{T_{CR}}$ and $\bar{E_{\text{coord}}}$ increase proportionally to the frame length in RFSA and FSA. Indeed, even though the value of *t_absorption_* tends to a constant value when the frame length increases, a higher number of slots per frame yields a greater delay. Since $\bar{T_{CR}}$ in Equation (12) is proportional to *t_absorption_* (constant) and to the frame length (increasing), the value of $\bar{T_{CR}}$ increases linearly with the frame length, as shown in Figure 4a. In addition, the coordinator has to listen to the channel in every frame for longer periods of time, yielding greater energy consumption.

Therefore, the frame length has to be tuned in RFSA and FSA to its optimum value, depending on the number of end-devices, taking into account that: (i) a frame length greater than the optimum has a cost in terms of average delay and energy consumed by the coordinator; and (ii) both the average delay and energy consumption increase dramatically when the frame length is much lower than the optimum value. Consequently, since the increase in delay and energy after the optimal point show a very small slope, when the number of end-devices is not known *a priori*, it is better to over-dimension the frame length in order to guarantee that the delay and energy consumption are kept low. Of course, synchronization issues may arise when the frame length is very long. This deserves further research to improve the accuracy of the internal clocks in wireless transceivers so that very long frames can be executed.

**Figure 5.**
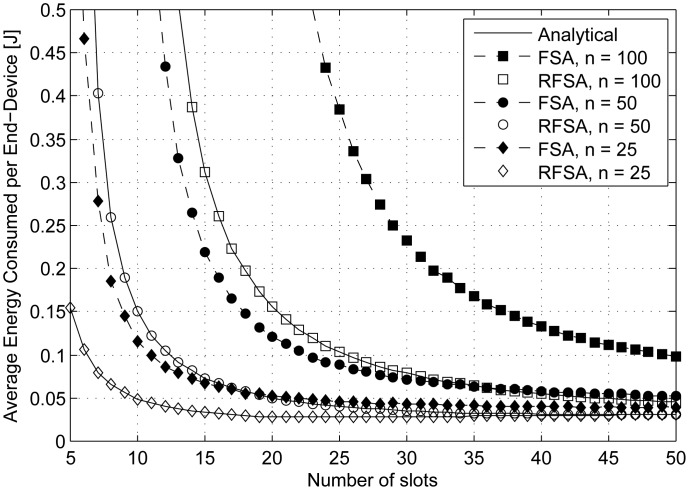
Average energy consumed per end-device in a data collection round over the frame length.

The average energy consumed per end-device in a data collection round $\left(\bar{E_{1-ed}}\right)$ is represented in Figure 5 as a function of the frame length (from five to 50 slots) for RFSA and FSA considering *n* = 25, 50 and 100 end-devices. As could be expected, the average energy consumed per end-device using RFSA and FSA tends to infinity when the frame length is very small. Indeed, the more frames required to complete a data collection round, the higher the energy consumed per end-device. In addition, the average energy consumed per end-device in FSA is always greater than in RFSA. This is due to the fact that in FSA, an end-device has to contend independently to transmit each one of its *L* data packets, and contrarily, in RFSA, an end-device has only to contend to transmit its first data packet and uses a reserved slot to transmit its other *L* − 1 packets, yielding less energy consumption per end-device. Results show that the average energy consumed per end-device tends to a minimum value when *m* ≥ *n*/2 and *m* ≥ *n* using RFSA and FSA, respectively. Indeed, although a longer frame length leads to longer standby and sleep periods in the end-devices, the use of low power standby and sleep modes (as shown in Table 1) yields reduced energy consumption. Therefore, the frame length has to be adjusted according to the number of contending end-devices in order to minimize the average energy consumed per end-device.

### 6.3. Delay and Energy Performance over the Number of End-Devices

In this section, we evaluate and compare the performance of RFSA and FSA, in terms of delay and energy consumption, with different numbers of end-devices in dense M2M networks.

**Figure 6.**
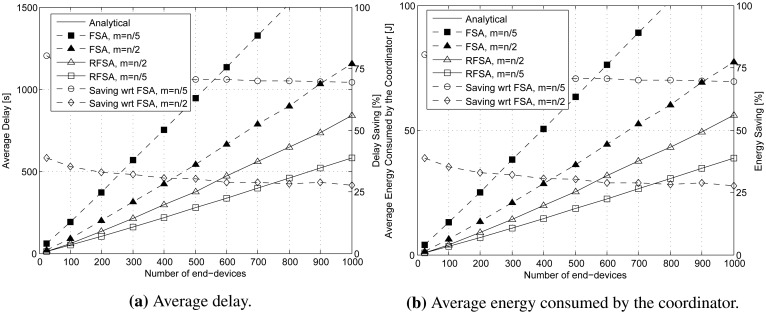
(**a**) Average delay (left vertical axis) to terminate a data collection round using *m* = *n*/2 and *m* = *n*/5 slots per frame; and (**b**) average energy consumption (left vertical axis) of the coordinator in a data collection round using *m* = *n*/2 and *m* = *n*/5 slots per frame. The delay and energy savings provided by RFSA with respect to FSA are shown in the right vertical axes.

The average delay and the average energy consumed by the coordinator in a data collection round using RFSA and FSA are represented in the left vertical axis of Figure 6, respectively, over the number *n* of end-devices (from 25 to 1000 end-devices). We have considered two different cases in both access protocols: (i) when the frame length is lower than the total number of end-devices and close to the optimal point that minimizes the delay and the energy consumption of the coordinator in FSA (*i.e.*, *m* = *n*/2); and (ii) when the frame length is close to the optimal in RFSA (*i.e.*, *m* = *n*/5).

As can be observed, the average delay and the average energy consumed by the coordinator using RFSA and FSA increase linearly with the number of end-devices, and as could be expected, the delay and the energy consumption are much higher using FSA than RFSA. The delay reduction and energy saving provided by RFSA with respect to FSA are shown on the right vertical axis of Figure 6, respectively. When *m* = *n*/2, RFSA provides savings of 25–30% with respect to FSA, and these savings increase up to a 70% when *m* = *n*/5. Indeed, the delay reduction and energy saving provided by RFSA are higher when its optimum frame length is used.

**Figure 7.**
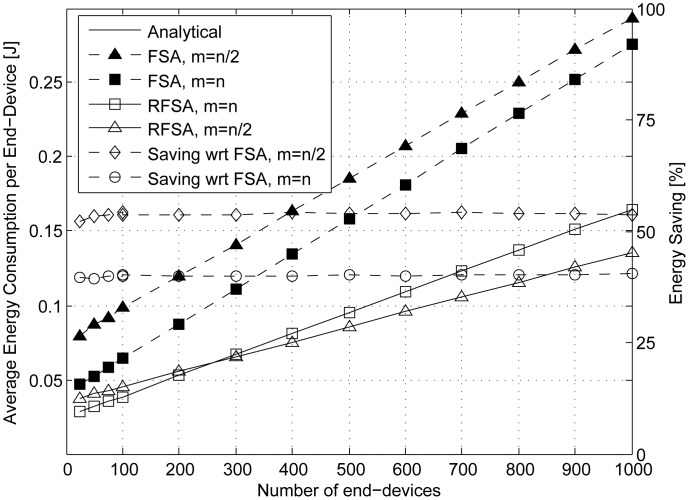
Average energy consumed (left vertical axis) per end-device in a data collection round using *m* = *n* and *m* = *n*/2 slots per frame. The energy savings provided by RFSA with respect to FSA is shown in the right vertical axis.

The average energy consumed per end-device in a data collection round using RFSA and FSA is represented in the left vertical axis of Figure 7 over the number *n* of end-devices (from 25 to 1000 end-devices). We have considered two different cases in both access protocols: (i) when the frame length is equal to the number of end-devices (*i.e.*, *m* = *n*); and (ii) when the frame length is lower than the total number of end-devices and close to the minimum value that guarantees that the energy consumed per end-device in RFSA is minimum (*i.e.*, *m* = *n*/2).

As can be observed, the average energy consumed per end-device using RFSA and FSA increases linearly with the number of end-devices, and as could be expected, the energy consumption is much higher using FSA than RFSA. The energy savings provided by RFSA is shown on the right vertical axis of Figure 7. When *m* = *n*, RFSA provides an energy savings of a 40% with respect to FSA, and it increases up to a 55% when *m* = *n*/2.

## 7. Conclusions

In this paper, we focus on M2M networks for data-collection applications where hundreds of end-devices periodically transmit data bursts to an M2M gateway upon request. The frame slotted ALOHA (FSA) protocol has been identified in the past as a good approach for data collection, due to its simplicity and good performance when every end-device has exactly one data packet to transmit. In this work, we propose the use of reservation FSA (RFSA) for M2M networks, where the end-devices generate fragmented data messages that consist of *L* packets of fixed length and *L* is an exponentially-distributed random variable. We have proposed a theoretical model to calculate the average delay and energy consumption for the RFSA protocol. Analytical and simulated results show that there exists an optimum frame length *m* that minimizes the value of the average delay and energy consumed by the gateway. This optimum value depends on the number *n* of end-devices, being *m* ≃ *n*/5 for RFSA and *m* ≃ *n*/2 for FSA. Results also show that the energy consumed per end-device tends to a minimum value when *m* ≥ *n*/2 and *m* ≥ *n* using RFSA and FSA, respectively. Existing algorithms for the estimation of the number of end-devices could be executed by the coordinator in order to adjust the frame length periodically to its optimal value periodically. The average delay and the average energy consumption in RFSA and FSA increase linearly with the total number of end-devices. RFSA outperforms FSA in terms of delay and energy consumption. In particular, the results show that RFSA can reduce the average delay and the coordinator's energy consumption by more than 25% with respect to FSA and the energy consumed per end-device by more than 50%. Therefore, the use of RFSA can considerably improve the energy efficiency of M2M networks. Future work aims at the design of an innovative reservation frame slotted-ALOHA protocol with dynamic adaptation in which the number of slots per frame can be dynamically adjusted to have a variable number of contention slots and reserved slots.

## Figures and Tables

**Table 1.** System parameters. FBP, feedback packet.

**Parameter**	**Value**	**Parameter**	**Value**
MAC header	8 bytes	Data-rate	250 kbps
Data payload	114 bytes	FBP payload	*m*·2 bits/slot
*T_preamble_*	160 μs	*T_IFS_*	192 μs
*T_SLOT_*	4.1 ms	*T_FBP_*	FBP length / 250 kbps
*ρ_tx_*	100.8 mW	*ρ_stby_*	525 μW
*ρ_rx_* = *ρ_σ_*	66.9 mW	*ρ_sleep_*	90 nW
